# Is Strain Elastography (IO-SE) Sufficient for Characterization of Liver Lesions before Surgical Resection—Or Is Contrast Enhanced Ultrasound (CEUS) Necessary?

**DOI:** 10.1371/journal.pone.0123737

**Published:** 2015-06-26

**Authors:** Ernst Michael Jung, Natascha Platz Batista da Silva, Wolfgang Jung, Stefan Farkas, Christian Stroszczynski, Janine Rennert

**Affiliations:** 1 University Hospital Regensburg, Department of Radiology, Regensburg, Germany; 2 University Hospital Regensburg, Department of Surgery, Regensburg, Germany; 3 Saarland University, Department of Materials Research, Saarbrücken, Germany; University of North Carolina School of Medicine, UNITED STATES

## Abstract

**Aim:**

To evaluate the diagnostic accuracy of IO-SE in comparison to IO-CEUS for the differentiation between malignant and benign liver lesions.

**Material and Methods:**

In a retrospective diagnostic study IO-CEUS and SE examinations of 49 liver lesions were evaluated and compared to histopathological examinations. Ultrasound was performed using a multifrequency linear probe (6–9 MHz). The loops of CEUS were evaluated up to 5 min. The qualitative characterization of IO-SE was based on a color coding system (blue = hard, red = soft). Stiffness of all lesions was quantified by a specific scaling of 0–6 (0 = low, 6 = high) using 7 ROIs (2 central, 5 peripheral).

**Results:**

All malignant lesions displayed a characteristic portal venous washout and could be diagnosed correctly by IO-CEUS. 3/5 benign lesions could not be characterized properly either by IO-CEUS or IO-SE prior to resection. Thus for IO-CEUS sensitivity, specificity, positive and negative predictive value and accuracy were 100%, 40%, 94%, 100% and 94%. Lesion sizes were between 8 and 59 mm in diameter. Regarding the IO-SE, malignant lesions showed a marked variability. In qualitative analysis, 31 of the malignant lesions were blue colored denoting overall induration. Thirteen malignant lesions showed an inhomogenous color pattern with partial indurations. Two of the benign lesions also displayed overall induration. The other benign lesions showed an inhomogenous color mapping. Calculated sensitivity of the SE was 70.5%, specificity 60%, PPV 94%, NPV 18.75%, and accuracy 69%.

**Conclusion:**

IO-CEUS is useful for localization and characterization of liver lesions prior to surgical resection whereas IO-SE provided correct characterization only for a limited number of lesions.

## Introduction

Studies have shown that contrast enhanced intraoperative ultrasonography (IO-CEUS) has a higher sensitivity for detecting malignant liver lesions compared to conventional intraoperative ultrasound (US) or pre-operative imaging modalities such as Computed tomography (CT) and Magnetic Resonance imaging (MRI) [1–3].

IO-CEUS may identify at least one additional malignant lesion in up to 30% of patients [4] causing a change of the therapeutic strategy in up to one-fifth to one-third of the patients [5,6].

IO-CEUS has also shown to be very useful for characterization of tumor size and morphology, particularly when using its dynamic assessment of the lesion’s microcirculation. Depending on the vascularization pattern from arterial to late phase, a differentiation of malignant from benign lesions is possible with satisfactory diagnostic accuracy [2].

The second generation US contrast agent SonoVue (Bracco, Milan, Italy)) consists of stabilized microbubbles that are composed of the hydrophobic gas sulphur hexafluoride (SF6) which is surrounded by a thin and flexible membrane of amphiphilic phospholipids. Thus, the gas within the microbubbles is stabilized but the shell remains flexible, allowing the microbubbles to change size and shape easily. These phospholipids serve as true blood-pool agents, which enable continuous real-time contrast sonography over an extended period of time. The microbubbles, have an average diameter of several micrometers, and are optimally oscillated when insonated with a low mechanical index (MI) technique. After being injected intravenously, the microbubbles are transported with the bloodstream and dispersed intravascularly [7]. Due to intraoperative ventilation, an early destruction of the microbubbles is likely to occur. Thus, repeated bolus injections of the contrast agent might be necessary [8].

Typical enhancement patterns for benign and malignant liver lesions were described in the 2012 EFSUMB guidelines regarding the use of CEUS for liver applications. For characterization of malignant liver lesions a wash out of the contrast agent beginning in the portal venous phase up to the late phase is a typical pattern besides showing an arterial irregular hyperenhancement. For benign liver lesions a continuous enhancement in the portal and late phases is typically observed. They can be further characterized by their enhancement patterns during the arterial phase. In most of the cases CEUS is also helpful for differentiation of atypical liver cysts [9].

Another approach for differentiating benign from malignant lesions is ultrasound elastography. The principle of quasi-static or strain elastography (SE) is based upon an assessment of the tissue deformation (strain) produced by an external palpation with the probe or by endogenous stress (e.g. cardiovascular movements) by following the way the speckle in the image moves, usually with a tracking algorithm working on the radiofrequency data. The data can be used to form an image that is coded in color or grey scale to show the pattern of strain, which is inversely related to the tissue stiffness and can be assessed subjectively. The images are semi-quantitative and do not directly depict the elasticity [10–12].

The technique relies on manual compression and color-coded depiction of lesions compared to the surrounding tissue. Thus, tissue stiffness relative to the surrounding tissue is estimated, which is widely consistent with the tissue composition and often modified by malignant or inflammatory diseases. Consistent with other cancers, malignant liver lesions are stiffer than the surrounding tissue, mostly due to the greater cell density. In contrast, benign lesions are usually softer and can display an inhomogeneous elasticity pattern. However, induration of the liver tissue–for example in cirrhotic liver diseases or following chemotherapy or deep-lying lesions–may limit the diagnostic accuracy of ultrasound elastography.

The aim of this study was to evaluate the diagnostic accuracy of intraoperative ultrasound elastography for differentiating between benign and malignant liver lesions compared to IO-CEUS and histopathology. Here, we performed for the first time a digital evaluation of the tumors compared to the surrounding tissue by Quantification-analysis (Q-analysis) of the strain elastography.

## Material and Methods

### Study design

From December 2010 until February 2012, 53 patients (33 male, 20 female, age 41–81 years, mean 62.4 years) with 60 suspected liver lesions were included in this retrospective study. Each patient underwent pre-surgical diagnostic computed tomography (CT) or magnetic resonance imaging (MRI) for detection and characterization of the liver lesions. Intraoperatively, each patient was examined using fundamental B- mode, Color Coded Doppler Sonography (CCDS), Power Doppler, Contrast Enhanced Ultrasound (CEUS) and strain elastography (SE). The use of CEUS for this study was approved by the local ethical committee (University of Regensburg). For SE, as an established diagnostic method without the use of contrast agent, no approval of the local ethical committee was required. Before the imaging procedures were conducted, written informed consent was obtained from each patient for MRI, CT and CEUS after the nature of the procedure was fully explained. Exclusion criteria of this study were contraindications for use of a contrast agent for CT or MRI, impaired renal function (creatinine >1.5 mg/dl, creatinine clearance < 30 ml/min), pre-existing strong allergic reactions and decompensated cardiac failure.

The indication for a surgical procedure was placed by an interdisciplinary tumor conference.

### Imaging techniques

#### ceMRI

MRI was performed on a 1.5-T whole-body scanner (Avanto, Siemens Medical Solutions, Germany) equipped with a high-performance gradient (Quantum) system (maximum gradient strength: 30 mT/m; slew rate: 125 T/msec). A combination of the standard body phased-array coil with spine array coils was used for signal reception.

For MRI, 0.1 ml/kg body weight of the liver specific contrast agent Primovist (Bayer HealthCare AG, Germany) was injected intravenously. T1-weighted VIBE (Volumetric interpolated breath hold examination) transversal dynamic scans were acquired 20, 40, 120 and 600 sec. after application of Primovist.

#### ceCT

The CT diagnosis was based on a triphasic contrast enhanced protocol using a Dual Source scanner (SOMATOM Definition Flash, Siemens, Germany), slice thickness 5 mm axial and 3 mm coronal MPR). The contrast bolus consisted of 1ml/kg Ultravist 370 i.v. (Bayer HealthCare AG, Germany) using a bolus trigger technique. The arterial phase started with a delay of 20–40 sec., the portal-venous phase with a delay of 60–70 sec. and the late phase >120 sec. after reaching the threshold.

#### Basic ultrasound examination

Intraoperatively, the whole liver was mobilized and palpated by a surgeon followed by a basic ultrasound examination (B-mode) with an intraoperative hockey stick probe of 2–4 MHz (HITACHI, EUB 6500). As no probes for contrast enhanced ultrasound are available for this ultrasound machine, the ultrasound examinations were subsequently repeated by one experienced ultrasound examiner (more than 5000 examinations each year for more than 10 years) using two multifrequency linear transducers (6–9 and 6–15 MHz, LOGIQ E9, GE Healthcare) that are available for CEUS and SE.

**Fig 1:**
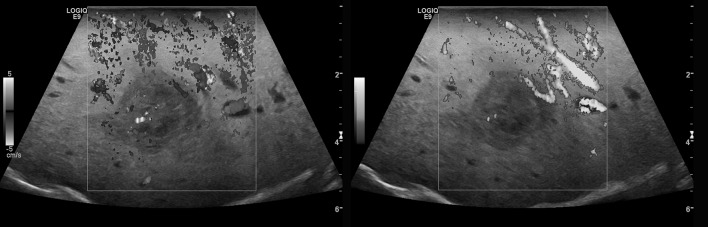
CCDS (left) and Power Doppler (right) for characterization of the vascularization of an HCC lesion.

First of all, a B-mode sonography in sweep technique of the whole liver was conducted. Color Coded Doppler Sonography (CCDS) and Power Doppler (PD) ultrasound were used to evaluate native vascularization (Fig 1). Flow parameters were adjusted to the lowest possible pulse repetition frequency (PRF < 1000 Hz) and the best possible color imaging without blooming artifacts.

#### IO-CEUS

**Fig 2:**
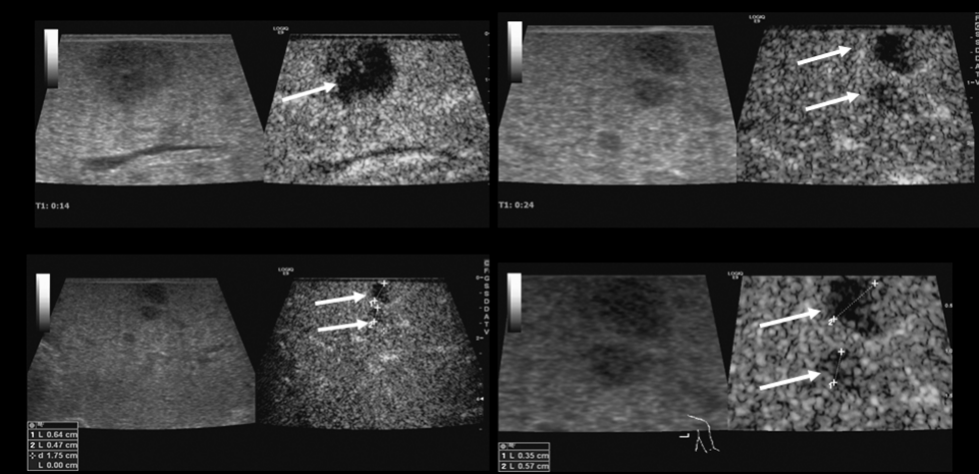
High resolution intraoperative contrast enhanced ultrasound (IO-CEUS) using a multifrequency linear probe (6–9 MHz) for detection of the primary tumor lesions and surrounding satellite lesions < 10 mm (arrow) in HCC.

The dynamic intraoperative contrast enhanced ultrasound (IO-CEUS) with bolus injections of 2.4 ml up to 5 ml of sulphur hexaflouride microbubbles (SonoVue, BRACCO, Italy) was conducted with a low mechanical index (MI < 0.16) applying CEUS with amplitude modulation and pulse inversion harmonic imaging (PIHI) technique (Fig 2). The contrast harmonic imaging technique (CHI) uses a contrast-specific detection mode for real-time evaluation of the contrast-agent enhancement. The complete data of the contrast-agent examination was recorded up to 5 min. The liver microcirculation was evaluated continuously from an early arterial phase (beginning 15 sec. after contrast application) until a late parenchymal phase (> 3 min.)

Again, an examination of the whole liver was performed using a sweep technique with digital storage of loops up to 5 min. (10–30 sec. per sweep). To reach the deeper layers of the liver using the harmonic imaging technique with CEUS it was necessary to use the 6–9 MHz linear probe for optimizing penetration.

#### SE

Following the IO-CEUS, strain elastography (SE) of the whole liver was performed. Five examinations were stored as digital cine loops up to 20 sec each. The stored elastogram was generated by a slight compression and decompression of the detected liver lesions. In addition, a quality marker for evaluation of the best compression mode was used. Consequently, only the highest image quality cine sequences were used for further quantification using the Q-analysis software which is integrated in the ultrasound machine. The aim of the quantitative analysis was to evaluate the color distribution numerically, thus providing more objective information. Q-analysis was performed by an independent reader.

**Fig 3:**
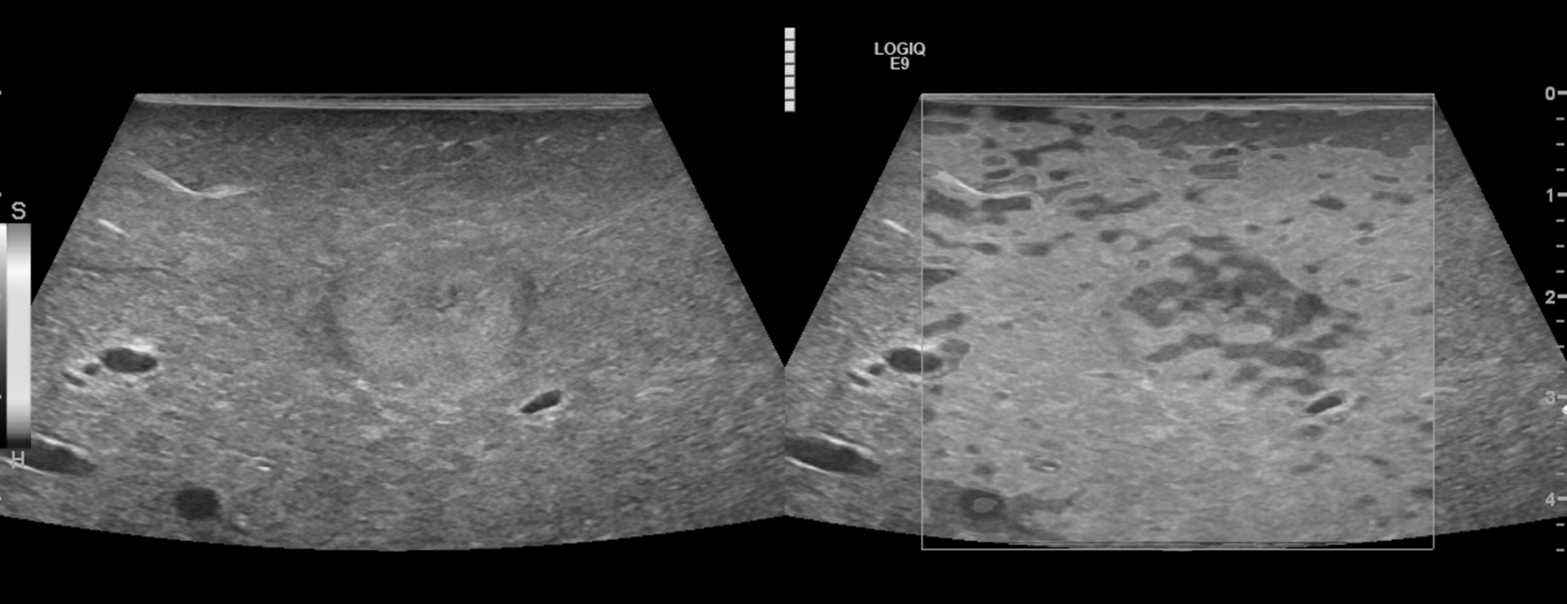
Intraoperative B-mode and strain elastography (SE) in a case of metastatic breast carcinoma. Displayed is a high quality image of SE with subsequent green quality bars. Echo inhomogeneous tumor lesion in B-mode (left), enhanced stiffness (dark gray) in SE (right) in comparison to the surrounding liver tissue (light gray).

The first step in analysis of the resulting elastography images was a qualitative characterization: the images were displayed using a 256-color map of strain using a scale from red (high strain, soft) to blue (low strain, hard) (Fig 3).

**Fig 4:**
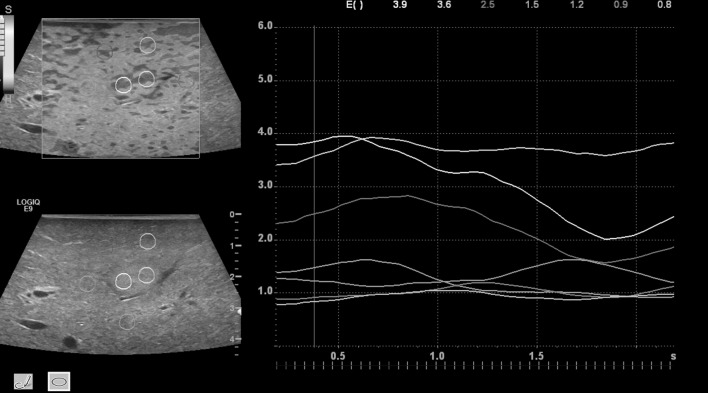
Q-analysis of SE of the tumor lesion. Two regions of interest (ROI`s) were placed in the center (turquoise, yellow), five ROIs in the surrounding liver tissue. Increased stiffness in the tumor center (level 4–5) in comparison to the liver tissue (level 1–3) (color coded line on left hand side). The lines document a continuous evaluation of the tissue stiffness over 10 seconds. High image quality was indicated by 5 green marks for SE.

The elasticity index reflects the color distribution within a defined region of interest (ROI) and was set as a scale from 0.0 (soft) to 6.0 (hard). Static ROIs were chosen to be round in shape. Two were placed in the center of the tumor (turquois and yellow) and five in the surrounding tissue (red, white, pink, orange, green), ranging from 5–10 mm in diameter (Fig 4). These were located at the same depth as their corresponding tumor ROIs.

US Elastography software measured the strain image or elastogram on which quantitative strain values could be assessed. The ROIs were placed on the color scale image for comparison of strain values of tissues with different elasticity. They were anchored to the target while moving along multiple frames to compensate for breathing or compression differences. The average strain values from these ROI’s were displayed as curves in a strain graph for each compression/decompression cycle.

The mean stiffness values of two ROI’s in the tumor center and five ROI’s in the surrounding liver tissue of the 60 lesions were analyzed.

### Image analysis

All MRI, CT, IO-CEUS and IO-SE examinations were analyzed by two experienced radiologists in consensus. For each modality used, each observer recorded the diagnostic findings. Furthermore, the image quality was documented on a four point scale: 1—excellent, 2—minor diagnostic limitations, 3 –major diagnostic limitations, 4—non-diagnostic.

Imaging modalities were evaluated using a picture archiving and communication system (Syngo Imaging; Siemens) and the data analysis hard-/software of the ultrasound system (LOGIQ E9, GE).

### Statistical analysis

At first an independent reading of the IO-CEUS and IO-SE digital stored images was performed on the DICOM reading system of the ultrasound machine (LOGIQ Works /GE). For data analysis, IBM-SPSS software (version 19.0, SPSS Inc., Chicago, USA) was used.

All results were presented as the mean ± standard deviation (SD). For calculation of the sensitivity, specificity, PPV, NPV and accuracy for detection of liver lesions for each modality the t- test for related samples was used.

We evaluated whether the pathological features and suspected diagnostic findings identified in the other imaging modalities (MRI, CT) could be confirmed using IO-CEUS. Additionally a semiquantitative evaluation of the color-coded images was performed for SE (blue: strong with homogeneous stiffness, green: particularly enhanced stiffness, yellow: moderate stiffness, red: reduced or less stiffness).

## Results

### Histopathology and tumor size

Out of the 53 patients, 54 malignant liver lesions were surgically removed, thus, a precise histopathological evaluation was possible. These malignant lesions included 18 hepatocellular carcinomas (HCC), 8 cholangiocellular carcinomas (CCC) and 28 metastases (colorectal carcinoma (21), neuroendocrine carcinoma (3), breast carcinoma (2), renal cell carcinoma (1), and malignant melanoma (1)).

Regarding the benign lesions, in 3 out of 6 cases an intraoperative biopsy was performed. Out of the 6 benign lesions two were found to be partially thrombosed hemangiomas, one a granuloma, one a dystrophic fibrosis and two were complicated, septated cysts. In the case of the atypical partially thrombosed hemangioma follow up by MRI showed no changes during the next 6 months. Two additional benign lesions were diagnosed correctly by IO-CEUS as complicated cysts. Thereby, an aspiration for cytopathology was conducted.

The tumor sizes of the 60 lesions ranged from 8 mm up to 59 mm, the mean size was 29 +/- 13 mm.

### Fundamental B-mode, CCDS and Power Doppler

50 mostly hypoechogenic, irregular tumor lesions were evaluated. Five notably hyperechogenic lesions showed marginal vascularization in CCDS and Power Doppler. The two complicated cystic lesions displayed no microvascularization.

### CEUS

**Fig 5:**
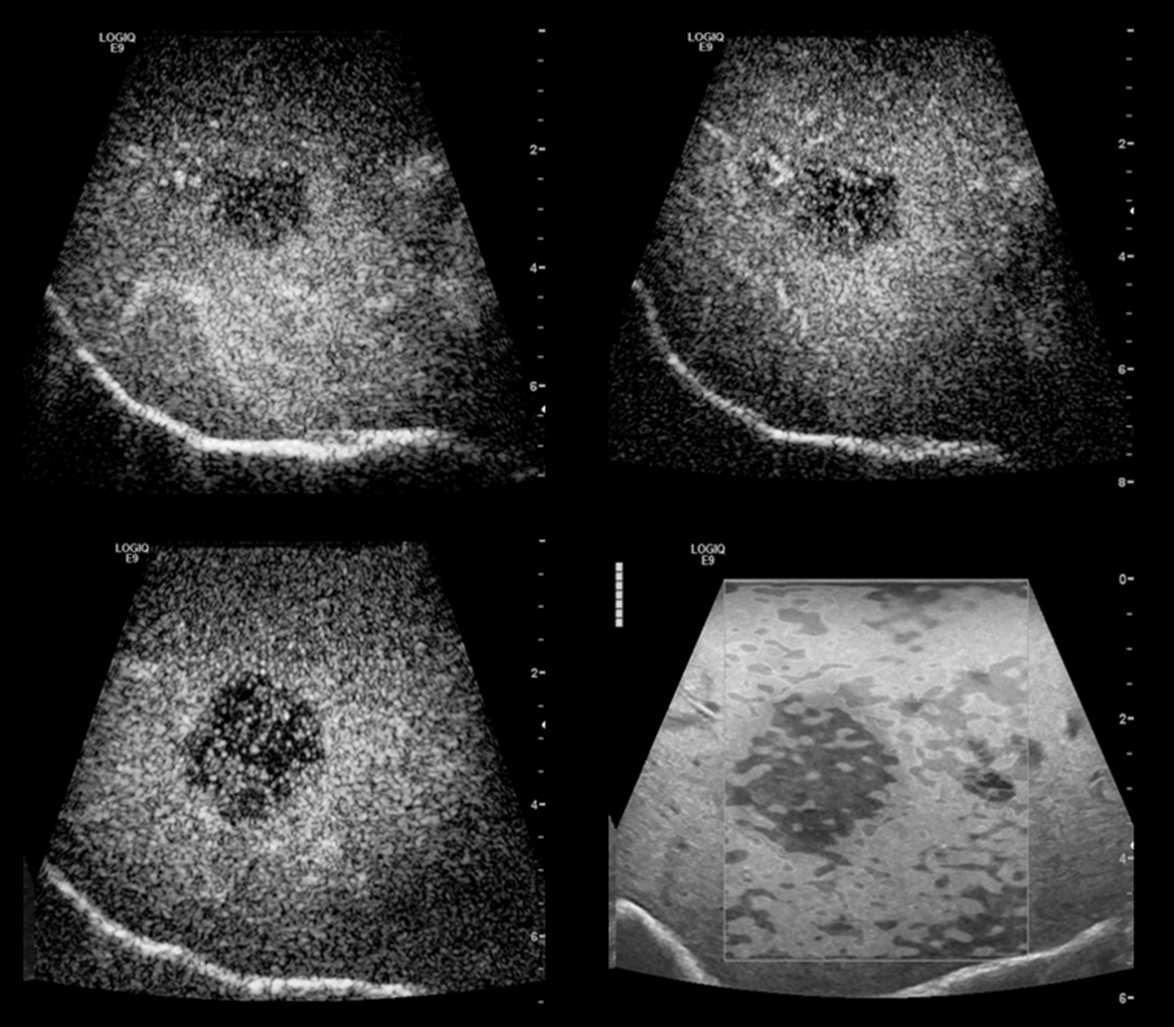
Comparison between intraoperative CEUS and SE in metastatic breast carcinoma. SE (d) displays a tumor lesion with increased stiffness, CEUS (a-c) of the same lesions showing wash out in the venous (40 seconds) (a) portal venous (70 seconds) (b) and late phase (2 minutes) (c).

All 54 malignant tumor lesions showed typical central or marginal arterial hypervascularization and wash out in the portal venous and the late phase (Fig 5).

As far as the benign lesions were concerned, the atypical thrombosed hemangiomas, the granuloma and the dystrophic fibrosis displayed a marginal arterial and prolonged parenchymal enhancement. The 2 complicated cysts showed irregular septal enhancement.

Using the t-test for related samples, calculation of the sensitivity, specificity, positive and negative predictive value and accuracy for IO-CEUS was 100%, 50%, 100%, 67.4% and 95%, respectively.

### SE

In 35 cases, semiquantitative evaluation of SE showed a homogeneous central area of complete blue coded stiffness in combination with an irregular margin and increased stiffness in comparison to the surrounding tissue. This, most importantly, depended upon the size of the liver lesion. Larger lesions often displayed central indurations due to tumor regression or previous chemotherapy, corresponding to increased stiffness.

Regarding the 18 HCC lesions, 2 cases were blue coded indicating increased stiffness. Twelve lesions showed moderate to high hardness values and 4 HCCs were found to be inhomogeneous, of moderate stiffness only. Six out of the 8 CCC lesions were blue coded, implying fairly stiff tumor tissue; the other 2 were of moderate stiffness. Ten of the 28 metastases were blue coded, 15 cases displayed moderate to high density values, and 3 cases were inhomogeneous, presenting with little to moderate stiffness only (Table 1).

**Table 1:** Fourfold table displaying the results of SE analysis compared to histopathology.

	SE malignant (stiffness scaling of 4–6)	SE benign (stiffness scaling of 0–3)
Histopathology malignant	38	16
Histopathology benign	3	3

**Fig 6:**
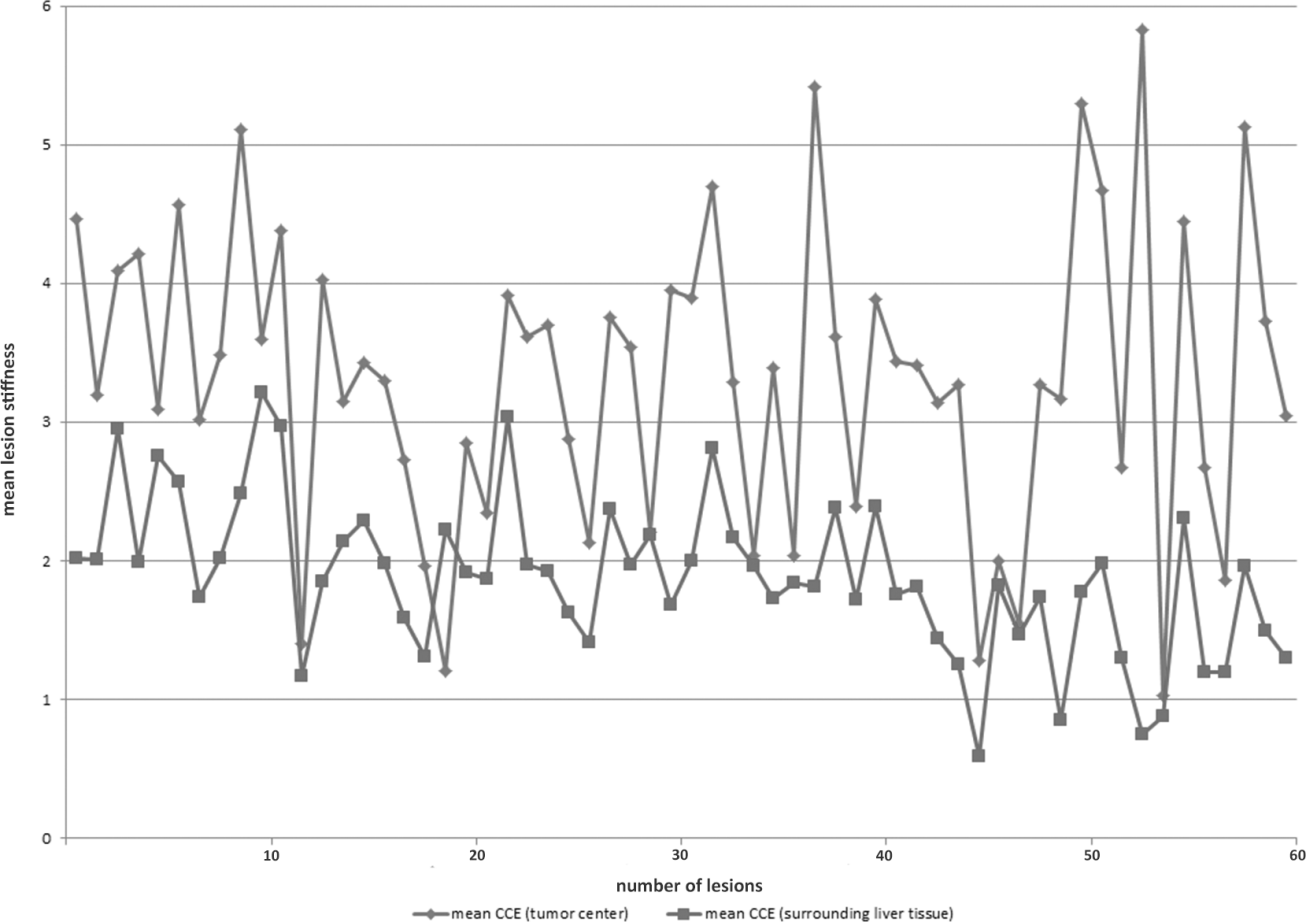
Evaluation of the tumor lesion stiffness by intraoperative SE. The y-axis represents the mean lesion stiffness on a scale from 0–6; the x-axis shows the number of lesions. Mean values by two regions of interest (ROIs) in the tumor center and five ROIs of the surrounding liver tissue of the 60 lesions. Blue lines show the mean results within the tumor lesion, red line within the surrounding liver tissue.

The mean of Q-Analysis values for the tumor tissue was 3.2 (standard deviation (SD) ± 1), the mean of the lesion surrounding tissue was 1.9 (SD ± 0.6) (Fig 6).

No homogenously blue coded area was found in the six benign lesions. One lesion expressed particularly high hardness values. In 3 other cases the lesion’s stiffness was considered moderate to low. An inhomogeneous morphology–as found in fibrotic and cystic benign or malignant lesions–was decisive for the color inhomogeneity of SE and the enhanced density values in the Q-analysis.

In conclusion the sensitivity of SE was 65%, specificity 25%. PPV was 73.7%, NPV 7.4%. The accuracy of intraoperative SE was 70%.

In all 53 patients (100%) SE and IO-CEUS were feasible. The image quality in all examinations was excellent or had only minor diagnostic limitations (1–2 in IO-CEUS SD ± 0.488, 1–2 in SE SD ± 0.383).

## Discussion

Surgical resection of liver tumors usually includes operative palpation followed by B-Scan ultrasound. When used by an experienced examiner, more lesions may be detected compared to pre-operative imaging method. However, B-Scan morphology only allows a limited differentiation of benign versus malignant lesions. Earlier studies revealed that CEUS—particularly the dynamic evaluation of the microvascularization–can do two things: i) it is a tool for the detection of small tumor lesions with a diameter of less than 12 mm and ii) allows a differentiation of benign from malignant lesions with high reliability [3,4,6,13]. Thus, dynamic evaluation of the microvascularization of tumor lesions is of great significance.

Current studies analyzing liver lesions using CEUS compared to contrast enhanced computed tomography (ceCT) and contrast enhanced Magnetic resonance imaging (ceMRI) have shown that malignant lesions, such as hepato-cellular carcinoma (HCC) or cholangio-cellular carcinoma (CCC), are characterized by an irregular arterial vascularization followed by wash-out in the late phase [14].

Recent studies have shown that not only the accuracy but also sensitivity and specificity regarding the differentiation of benign vs. malignant liver lesions were higher for IO-CEUS compared to CEUS, CT or MRI. This might cause further modifications of planned surgical management in up to a third of the patients [13,15–17].

Compared to other imaging modalities, CEUS and IO-CEUS require specific technology and an experienced examiner. Also, digital storage of the data is necessary for off-line quantification of the tumor perfusion. An alternative for pre- surgical detection and characterization of liver lesions is B-mode sonography using Tissue Harmonic imaging and Speckle Reduction Imaging together with strain elastography. In breast examination, using SE, malignant lesions are identified by a more or less inhomogeneous elasticity whereas benign lesions display an inhomogeneous, but similar pattern of elasticity as the surrounding tissue. Furthermore, Q-analysis of elastography offers a possibility for a retrospective evaluation of IO-CEUS consolidations by comparing regions of interest (ROIs) within the tumor with the surrounding tissue.

In this study, for the first time we evaluated and characterized liver lesions using contrast-enhanced ultrasound and strain elastography with manual compression intraoperatively and compared both methods to the histopathological results. The diagnostic test criteria were calculated using quantitative data gathered after surgery. The aim of the study was, therefore, to analyze to what extent malignant and benign liver lesions differ regarding their stiffness in relation to the surrounding liver tissue using Q-analysis tools and regarding the microcirculation characterized using IO-CEUS. Also, we evaluated whether a definite characterization of the liver lesions was possible by ultrasound elastography alone or if CEUS as an additional method was required.

In comparison to fundamental B-mode SE could provide a more precise characterization of the tumor morphology by additional evaluation of the tumor stiffness compared to its surroundings. But our results indicate that in patients with liver cirrhosis or following previous therapy (e.g. transarterial chemoembolization or chemotherapy) there is hardly a difference in stiffness of the lesion’s tissue and the surrounding cirrhosis with regenerative nodules. So that detection and characterization of tumors using SE remains difficult. In addition, some benign lesions were stiffer in the tumor center and/or the margins, which, in cystic lesions is known to be caused either by bleeding or septations. In these cases the evaluation of the dynamic tumor microvascularization with CEUS enables a more precise characterization of the tumor morphology, a better detection of smaller lesions (< 12 mm) and also the detection of tumor necrosis in particular.

The fact that SE is not as reliable as CEUS regarding characterization and detection of liver lesions emphasizes the necessity of high resolution IO-CEUS. Especially, when partially necrotic benign lesions, septated complicated cysts or regenerative dysplastic nodules are present. Thus, more studies with larger numbers of patients are required.

However, CEUS needs to be performed by an experienced examiner and requires high resolution US technology which is not always available.

Another limitation of our study is the restricted number of patients included, which can be explained by the fact that the combination of IO-SE and IO-CEUS is rather time-consuming (up to 10 min.), though CEUS could often realize a fast characterization of these liver lesions as malignant or benign, also without a biopsy. So, a more target-oriented surgical procedure is possible [1]. In addition, when a lesion was clearly benign no surgical resection would have been performed.

Furthermore, an examination using linear probes requires an additional mobilization of the liver segments close to the diaphragm. For this particular ultrasound technique, too, an experienced examiner is needed. Another important limitation of the study was that no standardized performance of the IO-SE was possible. Only a quality indicator of five green marks at maximum could be used for documentation of an equal image quality in each US examination. In addition the SE technology with Q-analysis is also not always available.

It is the combination of IO-SE and IO-CEUS which provides new possibilities for the assessment of tumor microvascularization and necrosis, especially when linear probes up to 15 MHz are used. This was first demonstrated in an experimental study [18]. Intraoperative SE and CEUS also offer alternatives for the monitoring of local ablative interventions of liver lesions [19].

In conclusion it can be said that IO-CEUS is a useful tool for localization and characterization of liver lesions prior to surgical resection (accuracy 94%) whereas IO-SE provided correct characterization only for a limited number of lesions (accuracy 69%).
